# Supplementary material for: Functional and Biological Characterization of the LGR5Δ5 Splice Variant in HEK293T Cells
Source: Int J Mol Sci. 2024 Dec 14;25(24):13417. doi: 10.3390/ijms252413417 (PMC11678308; doi:10.3390/ijms252413417)
Supplement: Supplementary file 1 [file ijms-25-13417-s001.zip › ijms-3289137-supplementary.pdf]

Supplemental figures and data for :

Kappler, M.; Thielemann, L.; Glaß, M.; Caggegi, L.; Güttler, A.; Pyko, J.; Blauschmidt, S.; Gutschner, T.; Taubert, H.; Otto, S.; et al. Functional and Biological Characterization of the LGR5Δ5 Splice Variant in HEK293T Cells. *Int. J. Mol. Sci.* **2024**, *25*, 13417. https://doi.org/10.3390/ijms252413417

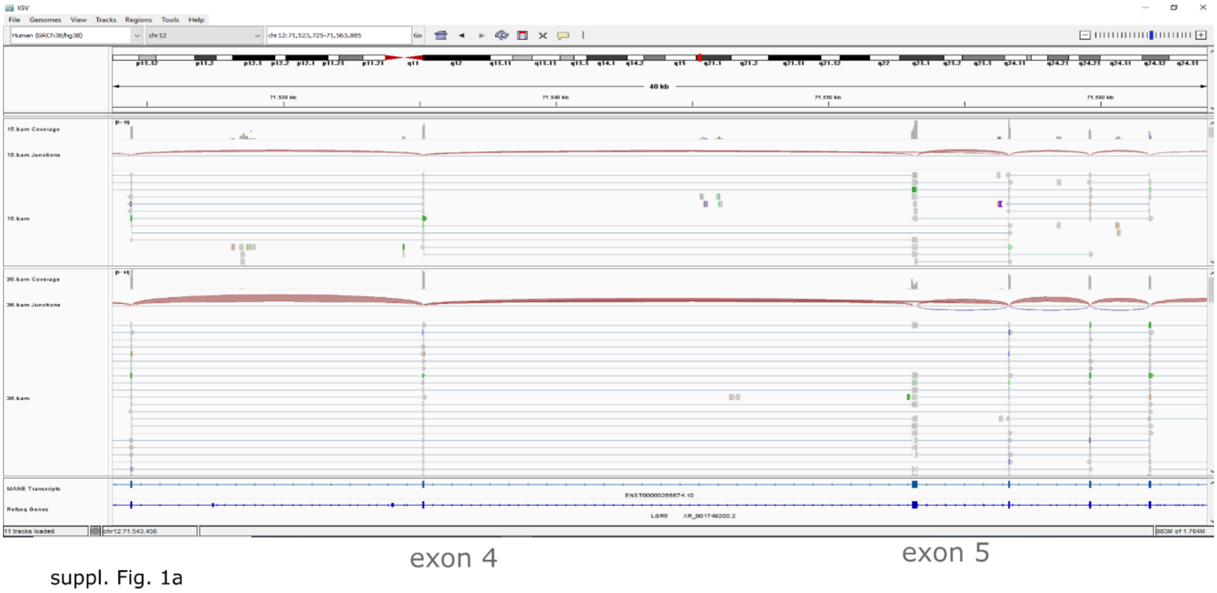

suppl. Fig. 1a

Figure S1 Sequence of LGR5FL and LGR5Δ5 in an unmodified HEK293T cell (1a)

Knockout procedure visualized (Created with BioRender.com).

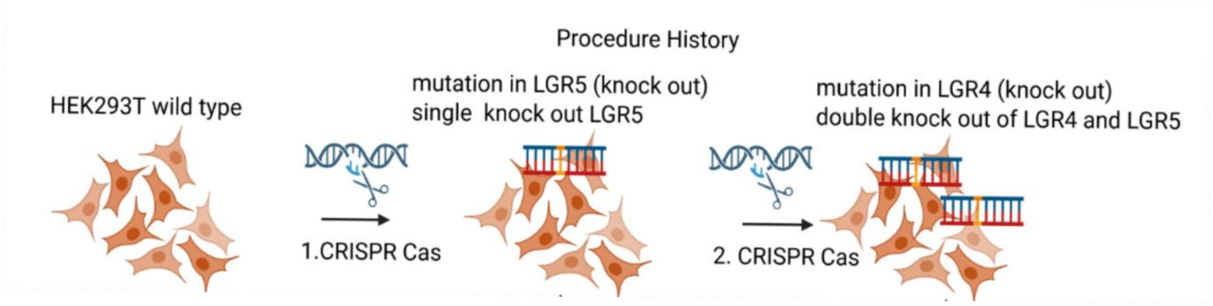

suppl. Fig. 1b

Figure S1 Procedure of the knockout of LGR5 and LGR4 (1b)

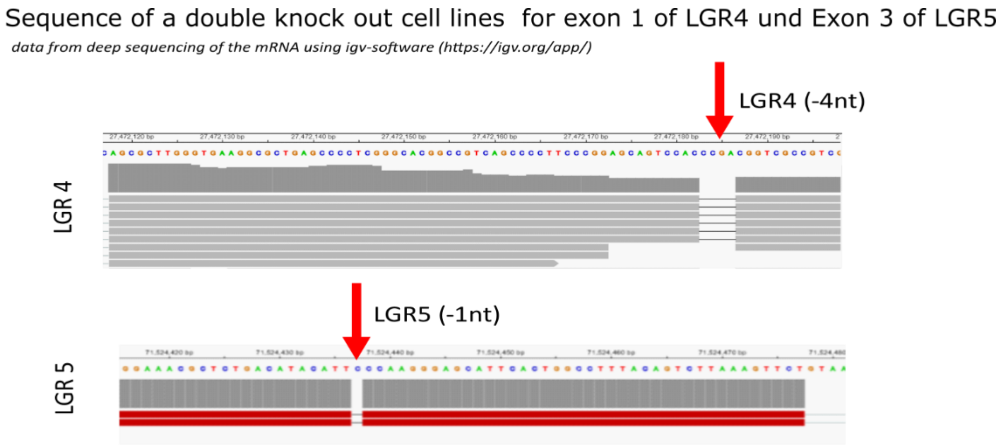

suppl. Fig. 1c

Figure S1 Sequence of a double-knockout [LGR4/5] HEK293T cell line with frameshift deletions in exon 1 (LGR4) and exon 3 (LGR5) (1c)

mRNA Sequence of a double knock out cell lines after rescue of LGR5 or LGR5Δ5 compared to empty vector conmtrol cells

data from deep sequencing of the mRNA using igv-software (https://igv.org/app/)

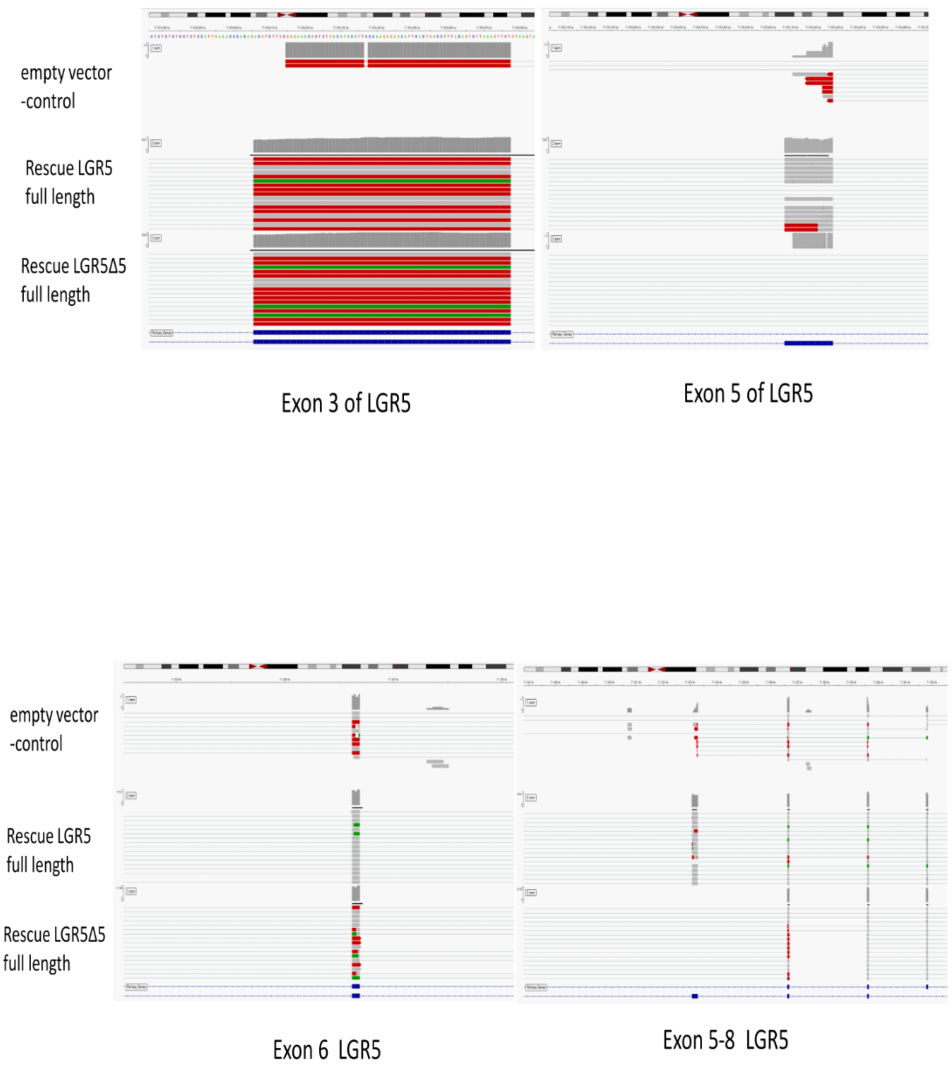

suppl. Fig. 2

Figure S2 Sequence of a double-knockout [LGR4/5] HEK293T cell line after the rescue of LGR5 or LGR5Δ5 compared with empty vector control cells (exons 3--8 of LGR5)

stable overexpression of LGR5FL or LGR5Δ5
after CRISPR Cas of only LGR4 or LGR4 and LGR5

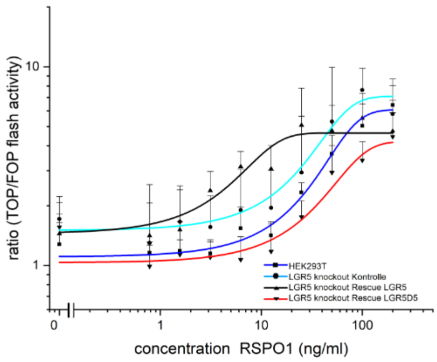

a

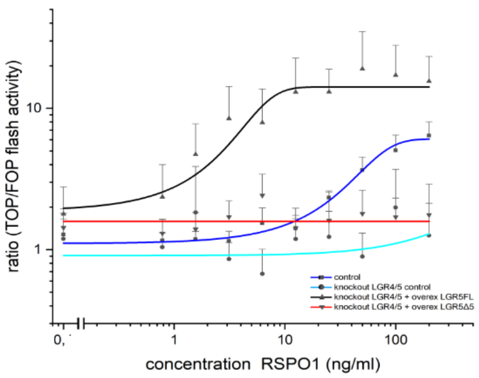

b

suppl Figure 3

Figure S3 TOPFlash assay to determine the influence of LGR5 modulation (LGR5 or LGR5Δ5 overexpression) on the Wnt pathway activity in CRISPR-knockout clones of a stable HEK293T cell line: a) an LGR5-knockout clone and b) an LGR4/5-double-knockout clone.

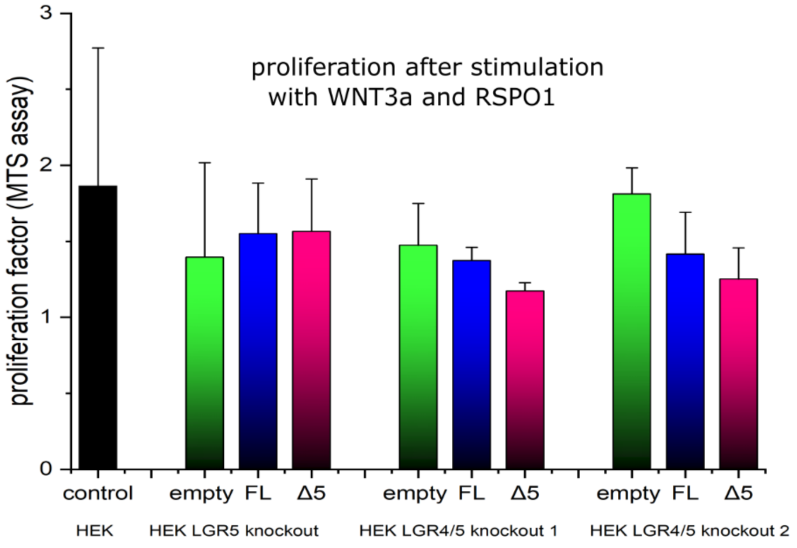

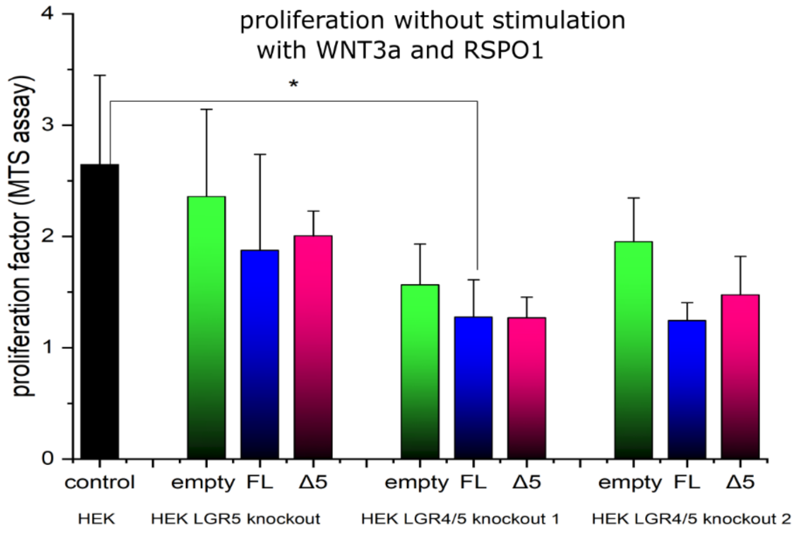

suppl. Figure 4

Figure S4 Effects of LGR5 modulation (LGR5 or LGR5Δ5 overexpression) on the proliferation of two HEK293T-LGR4/5 double-knockout cell lines, one single LGR5-knockout cell line and the control cell line HEK293T (control), with or without stimulation (HEK293T).

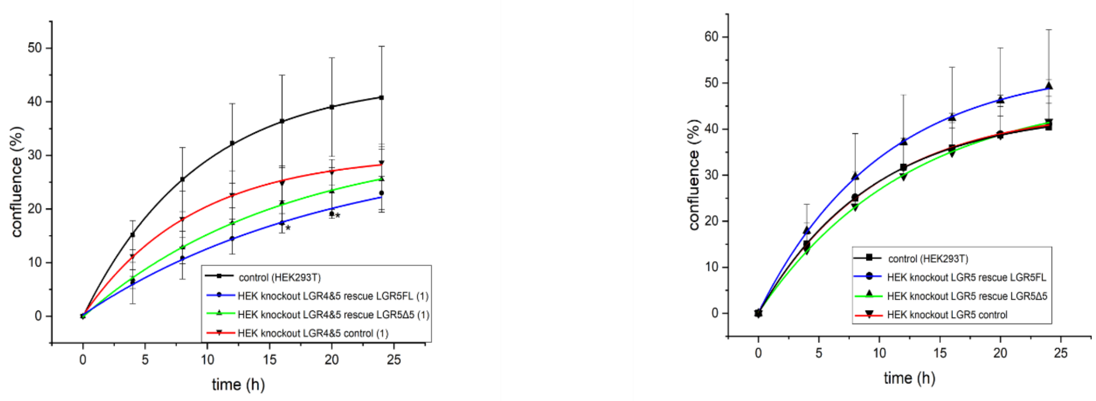

suppl. Figure 5

Figure S5 Analysis of migration in the scratch assay of one HEK293T-LGR5 knockout clone and a second HEK293/-LGR4/5 double knockout 1 clone with stable LGR5 or LGR5Δ5 expression compared with the control knockout (PLVX empty vector) cell line or unmodified HEK293T.

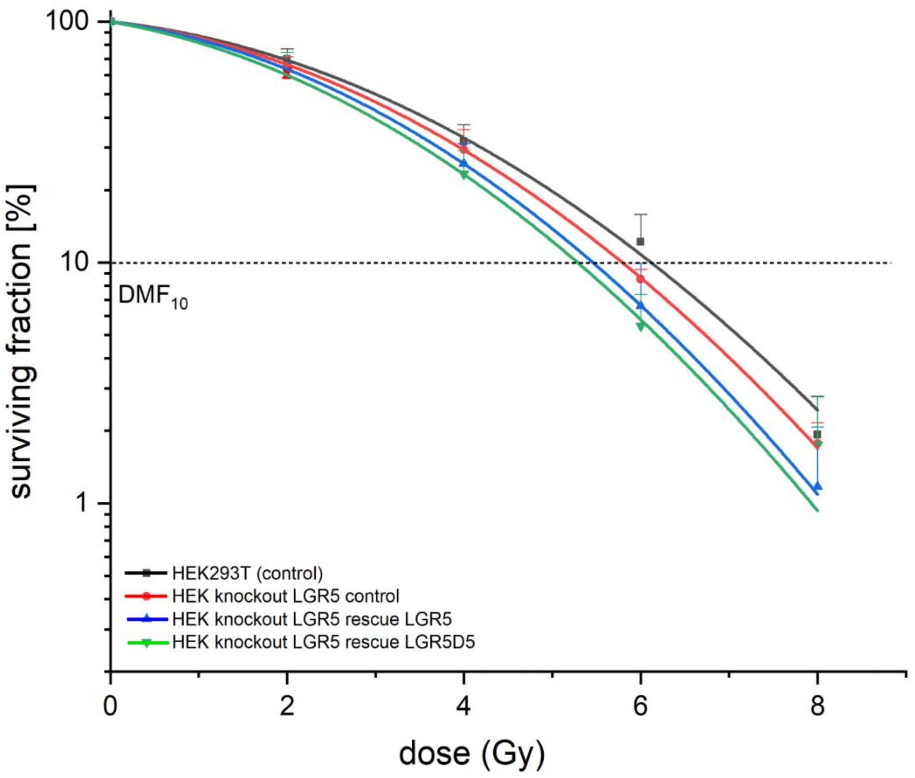

suppl. Fig. 6

Figure S6 Radiosensitivity of the unmodified HEK293T cell line and the HEK-LGR5 single knockout cell lines with stable LGR5FL or LGR5Δ5 expression or the empty vector expression cell line.

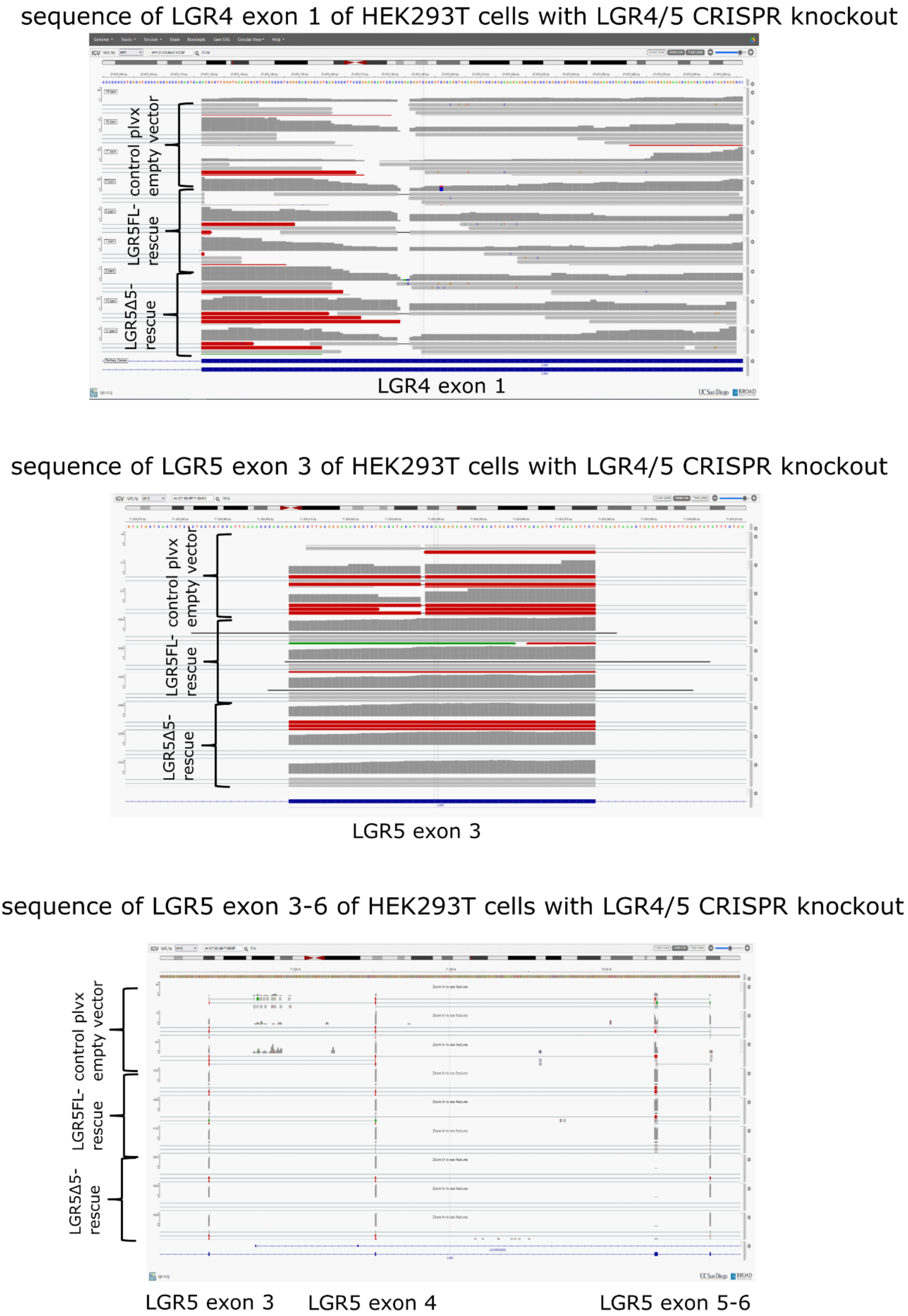

suppl. Fig. 7

Figure S7 Sequence of a double-knockout [LGR4/5] HEK293T cell line after the rescue of LGR5 or LGR5Δ5 compared with empty vector control cells

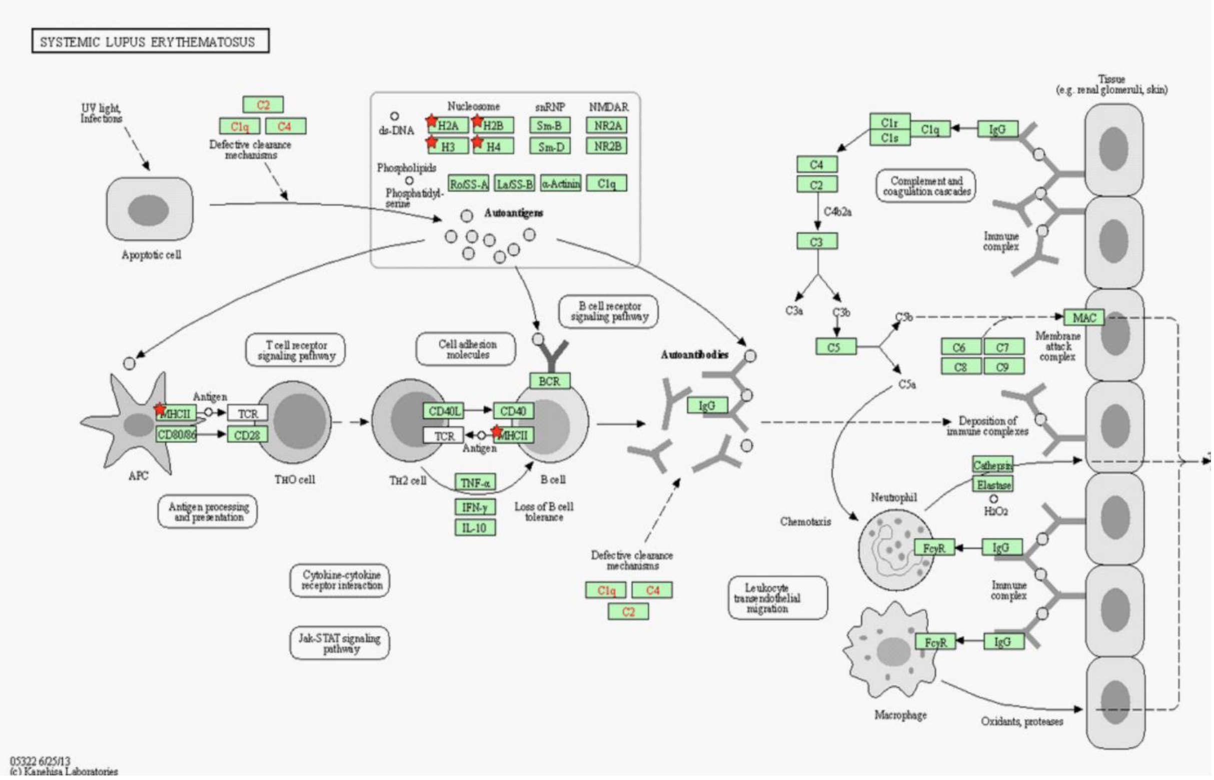

suppl. Fig. 8

Figure S8 Overrepresentation analysis of a double-knockout [LGR4/5]- and LGR5FL-overexpressing HEK293T cells identified a systemic lupus erythematosus pathway (david-data: https://davidbioinformatics.nih.gov/)

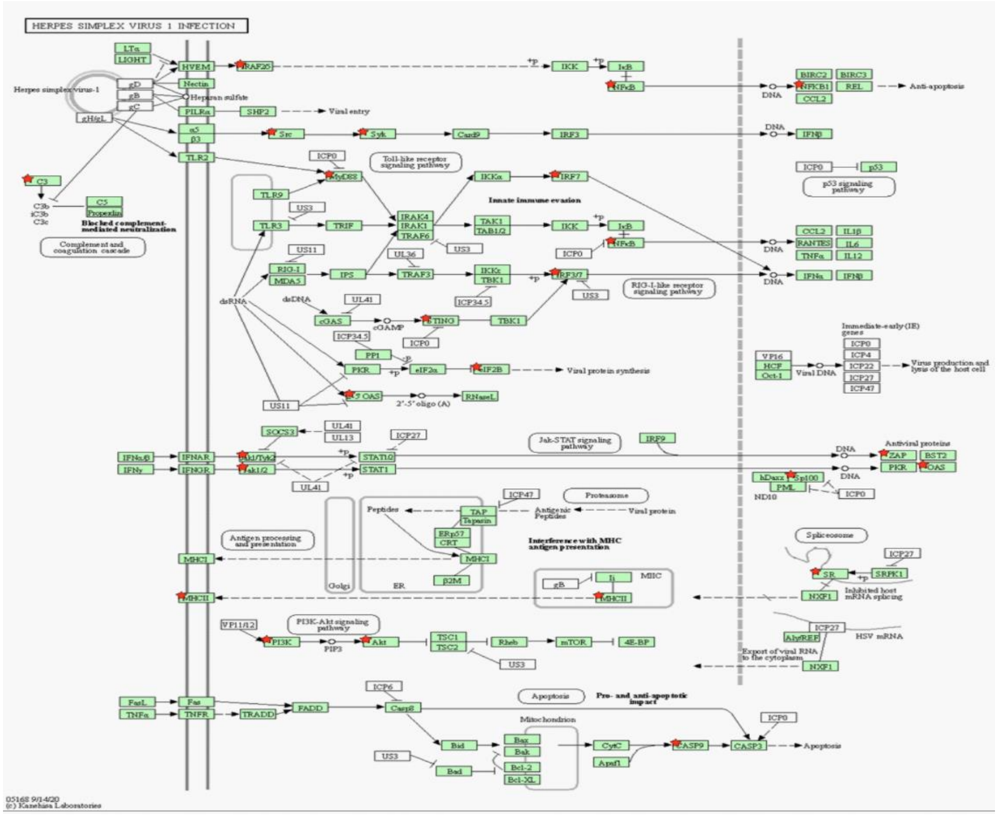

Nervous system development & Nervous system development
collagen type II alpha 1 chain(COL2A1)
ribosomal protein L22(RPL22)
collagen type IV alpha 1 chain(COL4A1)
EPH receptor B6(EPHB6)
nuclear cap binding protein subunit 2(NCBP2)
collagen type IV alpha 2 chain(COL4A2)
cullin 2(CUL2)
collagen type IV alpha 4 chain(COL4A4)
docking protein 6(DOK6)
collagen type V alpha 1 chain(COL5A1)
semaphorin 4D(SEMA4D)
LIM homeobox 2(LHX2)
collagen type VI alpha 2 chain(COL6A2)
dihydropyrimidinase like 3(DPYSL3)
adaptor related protein complex 2 subunit mu 1(AP2M1)
ephrin A1(EFNA1)
ephrin A3(EFNA3)
ephrin B2(EFNB2)
dystrophin related protein 2(DRP2)
ankyrin 1(ANK1)
myosin heavy chain 14(MYH14)
Rho guanine nucleotide exchange factor 7(ARHGEF7)
phosphatidylinositol-4,5-bisphosphate 3-kinase catalytic subunit beta(PIK3CB)
PDZ and LIM domain 7(PDLIM7)
lysophospholipase 2(LYPLA2)
ribosomal protein L26 like 1(RPL26L1)
ribosomal protein S6 kinase A1(RPS6KA1)

1p36 copy number variation syndrome & 1p36 copy number variation syndrome
delta like canonical Notch ligand 1(DLL1)
pseudouridine synthase like 1(PUSL1)
ceramide-1-phosphate transfer protein(CPTP)
transmembrane protein 52(TMEM52)
retention in endoplasmic reticulum sorting receptor 1(RER1)
peroxisomal biogenesis factor 10(PEX10)
hes family bHLH transcription factor 4(HES4)
interferon induced protein with tetratricopeptide repeats 1(IFIT1)
NOC2 like nucleolar associated transcriptional repressor(NOC2L)
sterile alpha motif domain containing 11(SAMD11)
hes family bHLH transcription factor 5(HES5)
mitochondrial ribosomal protein L20(MRPL20)
SSU72 homolog, RNA polymerase II CTD phosphatase(SSU72)
stromal cell derived factor 4(SDF4)
TNF receptor associated factor 5(TRAF5)
FA core complex associated protein 20(FAAP20)
ArfGAP with coiled-coil, ankyrin repeat and PH domains 3(ACAP3)
aurora kinase A interacting protein 1(AURKAIP1)
ubiquitin conjugating enzyme E2 J2(UBE2J2)
matrix remodeling associated 8(MXRA8)
integrator complex subunit 11(INTS11)
kelch like family member 17(KLHL17)
dishevelled segment polarity protein 1(DVL1)

suppl. Fig. 9

Figure S9 Overrepresentation analysis of single-knockout and LGR5Δ5-overexpressing HEK293T cells revealed herpes simplex virus 1 infection and neurogenesis or the 1p36 copy number variation syndrome pathway (david-data: https://davidbioinformatics.nih.gov/)

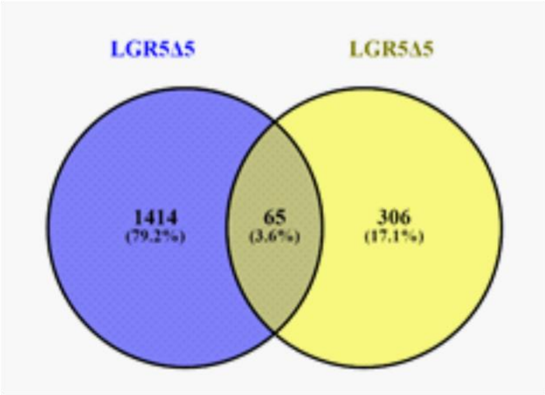

11 Cluster(s) Download File

Annotation Cluster 1		Enrichment Score: 2.31	G		Count	P_Value	Benjamini
☐	SMART	LamG	RT		4	2.1E-4	1.1E-2
☐	INTERPRO	Laminin G domain	RT		4	3.6E-4	5.1E-2

LamG & Laminin G domain
collagen type XI alpha 1 chain(COL11A1)
collagen type V alpha 1 chain(COL5A1)
growth arrest specific 6(GAS6)
cadherin EGF LAG seven-pass G-type receptor 3(CELSR3)

suppl. Figure 10

Figure S10 Venn diagram and overrepresentation analysis of single-knockout and double-knockout [LGR4/5] cells overexpressing LGR5Δ5 in HEK293T cells identified the LamininG pathway

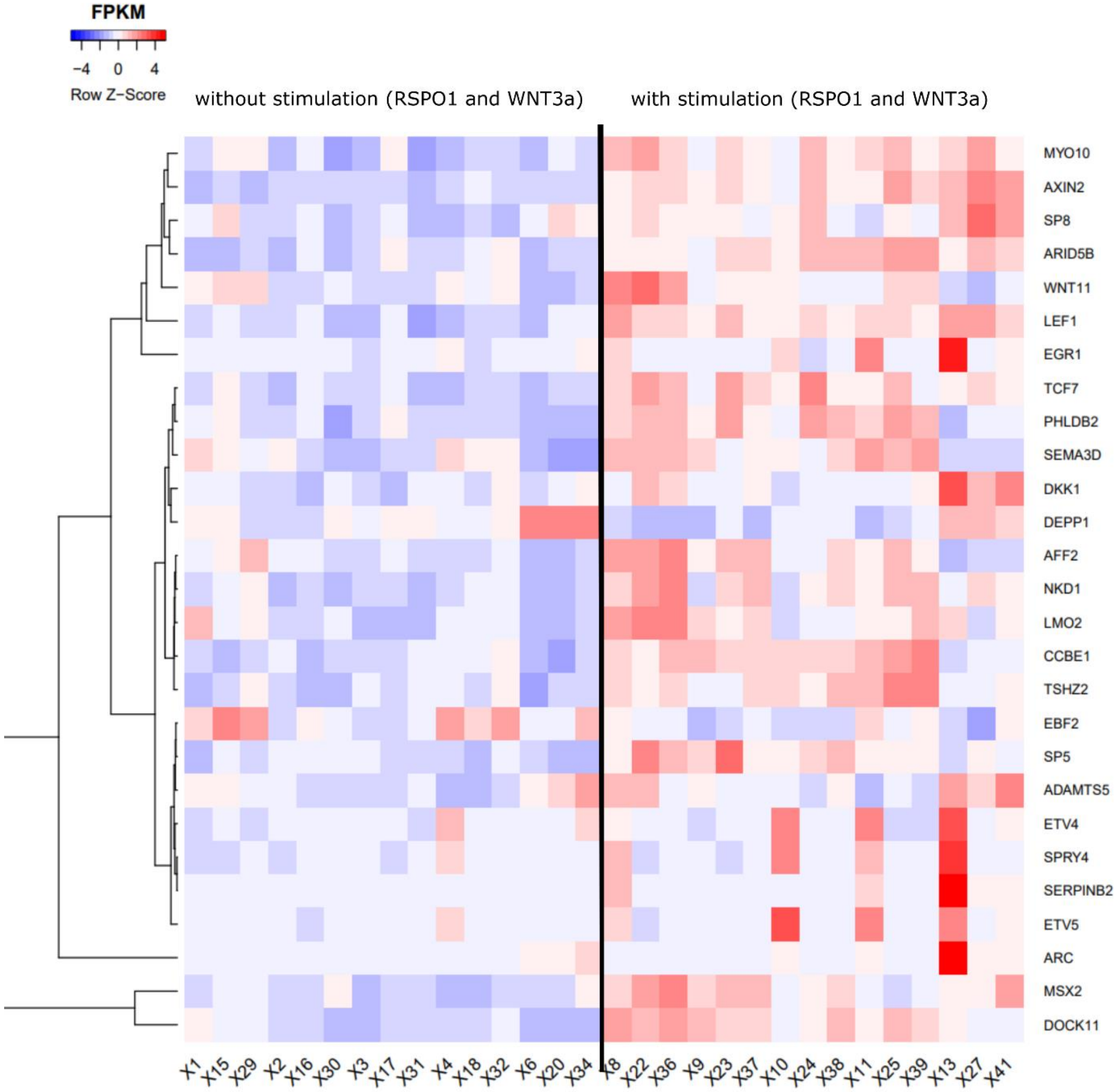

suppl. Figure 11

Figure S11 Heatmap of genes (n=27) significantly altered by stimulation with Wnt3A and RSPO1 (HEK293T cells, single LGR5 knockout cells with LGR5Δ5, LGR5FL rescue or empty vector control, and HEK-LGR4/5 knockout cells with LGR5FL rescue). (n=3 for each cell line, 15 unstimulated samples and 15 stimulated samples in a paired test system). (FDR≤0.05; |log2FC|≥0.5)

siRNA knock down of ZRFN3&RNF43 and overexpression of LGR5FL or LGR5Δ5

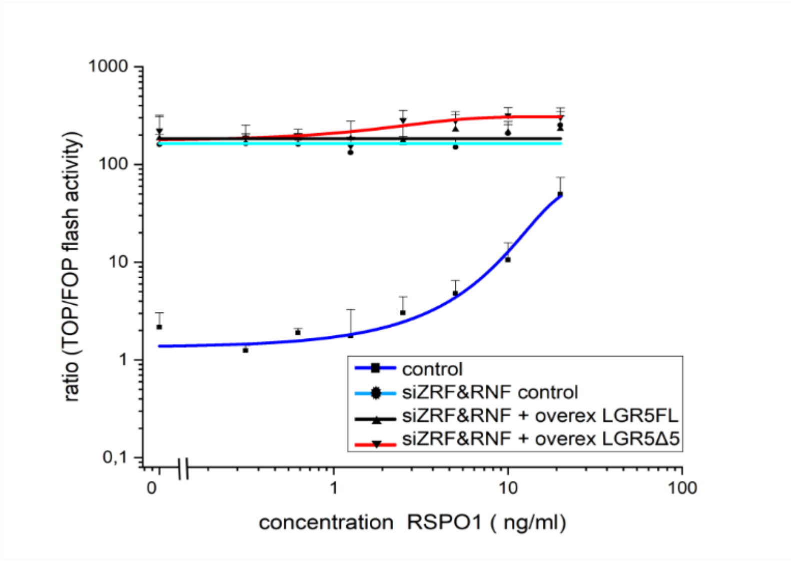

suppl. Fig. 12

Figure S12 TOP/FOP Flash Assay for measuring Wnt pathway activity in modified HEK293T cells after siRNA-mediated knockdown of ZRFN3 & RNF43 and overexpression of an empty vector (light blue line), LGR5FL (black line) or LGR5Δ5 (red line). The control (dark blue line) corresponds to the empty vector control of Figure 3a.

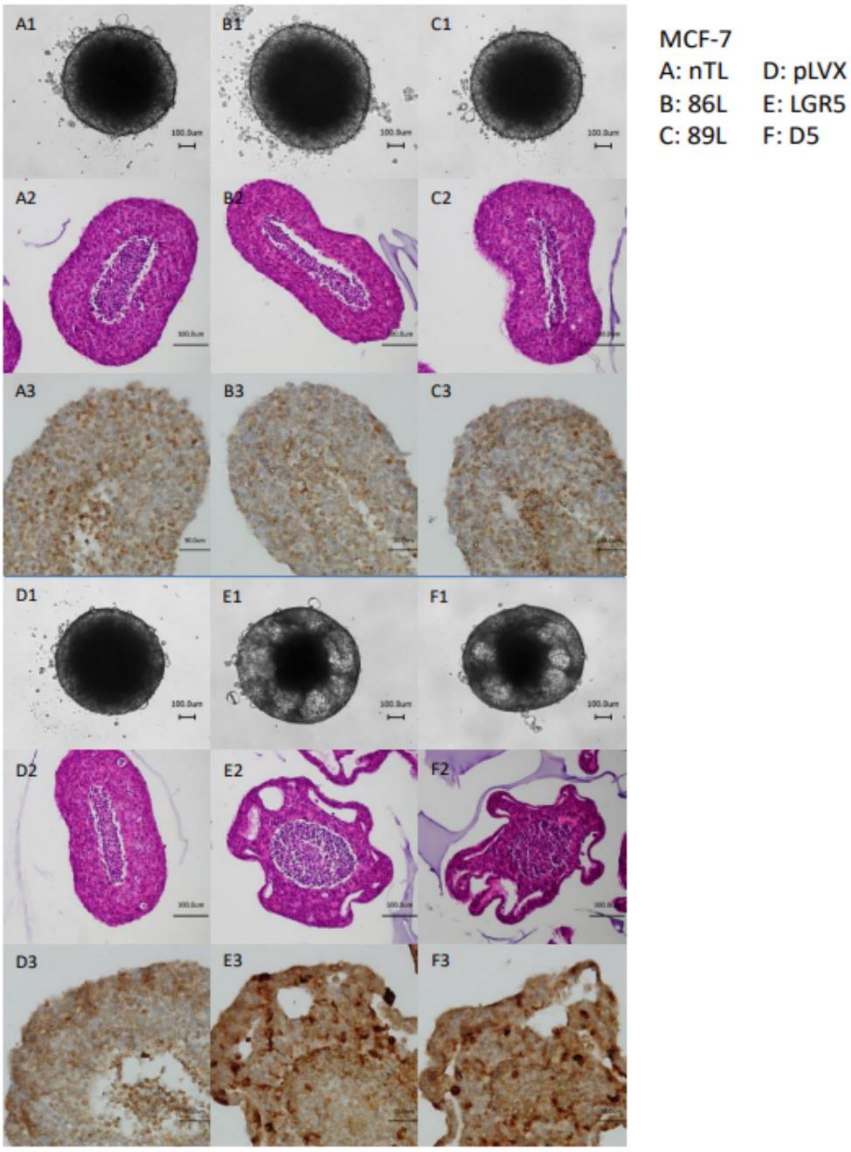

suppl. Fig. 13

Figure S13 Spheroids of the MCF-7 cell line (mammary tumor). The spheroid column (A1-F1) shows transmitted light images of the spheroids after seven days of cultivation and staining with hematoxylin–eosin (A2-F2) or LGR5 (A3-F3) after transduction with the shRNA (short hairpin) and LGR5FL or LGR5Δ5 expression plasmids [20]. nTL denotes the nontargeting shRNA control, and shRNA1 (86 L) and shRNA2 (89 L) are the two shRNAs used to reduce LGR5 expression [20]. LGR5FL or LGR5Δ5 denotes the transduction of the LGR5FL or LGR5Δ5 expression plasmids. plVX denotes the corresponding empty vector control. The marking at the bottom left corresponds to 100 μm, and that in the column LGR5 staining corresponds to 50 μm

Additional information regarding expression of specific genes

Expression of genes in the single-knockout cell line

ANGPT1 (Angiopoietin 1) was upregulated in single-knockout LGR5Δ5 cell lines (log2FC=+ 1.7), whereas COL11A1 (collagen alpha-1 (XI)) was downregulated (log2FC=- 2.2) in the [LGR5] single-knockout cell line. Moreover XYLT1 (xylosyltransferase 1) was also downregulated in the single knockout LGR5Δ5 cell lines (log2FC=-1.3) as well as FZD10 (Frizzled-10 (Fz-10)) in single knockout LGR5Δ5-overexpressing cell lines *(log2FC=-2.8)* (Table S3, 7).

ORA for LGR5FL in double knock out cells

Gene enrichment analysis (overrepresentation analysis (ORA)) for LGR5FL overexpression (203 genes) identified systemic lupus erythematosus (p<0.02)*-adjusted p value) (Figure S8) as a possibly associated related disease. Nine differentially expressed genes were responsible for the enrichment of this pathway, eight of which belong to the histone complex (H2B-H4 clustered histone enzymes) (Table S2, S5), and six out of them are also associated with TCF-dependent signaling in response to Wnt ((p<0.05)* (Table S5)). Most histone complex hits are also significantly associated with 1) HDACs deacetylating histones (p<0.03)* or 2) RNA polymerase I promoter opening (p<0.05)* (Table S5).

This interesting finding regarding the effect of LGR5FL modification on the posttranscriptional modification of proteins was supplemented by a second significantly identified functional cluster that included different collagen types (COL2A1, COL5A1, COL5A2, and COL11A1). These genes were identified in the category of fibrillar collagen (p<0.03) * and together with the histone cluster in the category of hydroxylation (p<0.001) * (Table S2, S5). LGR5FL overexpression resulted in the deregulation of only four zinc finger (ZNF) genes, which contrasts with LGR5Δ5 overexpression

For the single-knockout HEK293T cell lines (with LGR5 knockout and endogenous expression of LGR4) only 14 genes with found significantly associated with LGR5FL overexpression. Whereas LGR5Δ5 overexpression was significantly associated with deregulated of 1479 genes compared with empty vector control cells, respectively (Tables S6, S7). Gene enrichment analysis (overrepresentation analysis (ORA)) for LGR5Δ5 rescue revealed herpes simplex virus 1 infection (p<0.01*), neurogenesis (p<0.001*), or the 1p36 copy number variation syndrome pathway (p<0.001*) (Figure S9).

The comparison of LGR5FL overexpression in single- versus double-knockout cells is not shown because only 3 genes were found (Table S2 versus S6).

sgRNA	Sequenz (5`-3`)	core sequenz (5`-3`)	Gene	orientation	lokalisation	date	source	comment
LGR5 sgRNA_E3_F	CACCGCTCTGACATACATTCCCAA	GCTCTGACATACATTCCCAA	LGR5	sense	Exon 3	26.06.2017	sgRNA designer	luded Brunello and in TKC
LGR5 sgRNA_E3_R	AAACTTGGGAATGTATGTCAGAGC	TTGGGAATGTATGACAGAGC	LGR5	antisense	Exon 3	26.06.2017	sgRNA designer	luded Brunello and in TKC
LGR4 sgRNA_E5_F	CACCGCTGCGACGGCGACCGTCGGG	CTGCGACGGCGACCGTCGGG	LGR4	sense	Exon 1	27.10.2021	sgRNA designer (BROAD)	
LGR4 sgRNA_E5_R	AAACCCCGACGGTCGCCGTCGCAGC	CCCGACGGTCGCCGTCGCAG	LGR4	antisense	Exon 1	27.10.2021	sgRNA designer (BROAD)	

Suppl. Table S10
